# Targeted inhibition of STAT3 as a potential treatment strategy for atherosclerosis

**DOI:** 10.7150/thno.35528

**Published:** 2019-08-14

**Authors:** Qi Chen, Jianjun Lv, Wenwen Yang, Baoping Xu, Zheng Wang, Zihao Yu, Jiawei Wu, Yang Yang, Yuehu Han

**Affiliations:** 1 Key Laboratory of Resource Biology and Biotechnology in Western China, Ministry of Education. Faculty of Life Sciences, Northwest University, 229 Taibai North Road, Xi'an 710069, China; 2 School of Basic Medicine, The Fourth Military Medical University, 169 Changle West Road, Xi'an 710032, China; 3 Department of Cadio-Thoracic Surgery, Wuhan General Hospital of The People's Liberation Army, 627 Wuluo Road, Wuhan 430070, China; 4 Department of Cardiovascular Surgery, Xijing Hospital, The Fourth Military Medical University, 127 Changle West Road, Xi'an 710032, China

**Keywords:** atherosclerosis, STAT3, endothelial cell dysfunction, macrophage polarization, inflammation, immunity, inhibitors

## Abstract

Atherosclerosis is the main pathological basis of ischemic cardiovascular and cerebrovascular diseases and has attracted more attention in recent years. Multiple studies have demonstrated that the signal transducer and activator of transcription 3 (STAT3) plays essential roles in the process of atherosclerosis. Moreover, aberrant STAT3 activation has been shown to contribute to the occurrence and development of atherosclerosis. Therefore, the study of STAT3 inhibitors has gradually become a focal research topic. In this review, we describe the crucial roles of STAT3 in endothelial cell dysfunction, macrophage polarization, inflammation, and immunity during atherosclerosis. STAT3 in mitochondria is mentioned as well. Then, we present a summary and classification of STAT3 inhibitors, which could offer potential treatment strategies for atherosclerosis. Furthermore, we enumerate some of the problems that have interfered with the development of mature therapies utilizing STAT3 inhibitors to treat atherosclerosis. Finally, we propose ideas that may help to solve these problems to some extent. Collectively, this review may be useful for developing future STAT3 inhibitor therapies for atherosclerosis.

## 1. Introduction

Cardiovascular and cerebrovascular diseases, especially heart attack and stroke, significantly contribute to worldwide mortality 1, causing approximately 17 million deaths per year 2. Moreover, atherosclerosis is recognized as the original cause of most cardiovascular and cerebrovascular diseases 3-5. As a chronic progressive inflammatory arterial wall disease, atherosclerosis is characterized by the accumulation of lipids in the intima, thickening of the arterial wall, and narrowing of the vascular cavity. The major drivers leading to atherosclerosis include hyperlipidemia, hyperglycemia, insulin resistance, hypertension, and other factors such as genetics, age, cigarette smoking, and mental status 6. Thus, various atherosclerosis treatments have emerged that directly target the above risk factors, such as lipid-lowering medications and antiplatelet aggregation therapies. However, these treatments are not entirely effective due to incomplete knowledge of the mechanisms of and effective target sites for atherosclerosis. Therefore, the mechanism of atherosclerosis has been a popular focus of research, from which scholars hope to find novel breakthroughs, develop feasible intervention measures, and improve the overall prevention and treatment strategies for atherosclerosis. In addition, with the progress and development of basic and clinical research, study of the pathogenesis of atherosclerosis has made great strides. The pathological mechanisms of atherosclerosis are complex and can be mainly summarized as endothelial cell dysfunction, macrophage polarization, inflammation, and immune responses. Emerging studies reveal that the signal transducer and activator of transcription 3 (STAT3) may play a critical role in all these factors, which indicates that STAT3 might become a new target of atherosclerosis therapies.

STATs, consisting of seven members (STAT1, STAT2, STAT3, STAT4, STAT5a, STAT5b, and STAT6) 7-11, have dual functions in signal transduction and transcriptional regulation. STAT3, one of the seven STAT members, was initially identified by two individual groups in 1994 12, 13 and has increasingly gained focused attention due to its significant roles in diverse biological processes, including cell proliferation, cell differentiation, cell survival, inflammation, immunity, and angiogenesis 14. Since the gene Stat3 was first described as an oncogene in 1999 15, STAT3 has become the research focus for several disease areas as a potential anticancer target. Previous studies have verified that STAT3 plays an essential role in various diseases, including cancers 16, myocardial ischemic injury 17, stroke 8, and obesity 18. Recently, with the study of cardiovascular and cerebrovascular diseases becoming increasingly popular, STAT3 has been demonstrated to play roles in several cardiovascular diseases, including arteriosclerosis, cardiac hypertrophy, and heart failure 19-22. Although atherosclerosis is considered the critical pathological basis of most cardiovascular and cerebrovascular diseases, no specific reviews are aimed at the emerging roles of STAT3 in atherosclerosis. Thus, the present review aims to fill this gap.

In this review, by summarizing the current literature, we highlight the essential roles of STAT3 in atherosclerosis and present STAT3 inhibitors that may become potential treatment agents for atherosclerosis. First, we describe the general background of STAT3, including its structure, function, and regulation. Subsequently, we discuss the pathological roles of STAT3 in atherosclerosis from three independent but related biological processes, endothelial cell dysfunction, macrophage polarization, inflammation, and immunity. Moreover, we summarize the current inhibitors of STAT3 (Table 1) and explore their implications in atherosclerosis treatments. Finally, we highlight some potential issues and propose some solutions to these issues. In conclusion, this review may contribute to the application of STAT3 as a novel target of atherosclerosis therapies.

## 2. STAT3

### 2.1 The STAT and JAK families

Members of the STAT protein family are localized in the cytoplasm, can translocate into the nucleus to bind DNA, and dually function in signal transduction and transcriptional regulation. These proteins have been shown to participate in diverse cellular processes, including stem cell maintenance, lipid metabolism, neuron function, carcinogenesis, inflammation, and immunity 16, 23-26.

**Figure 1:**
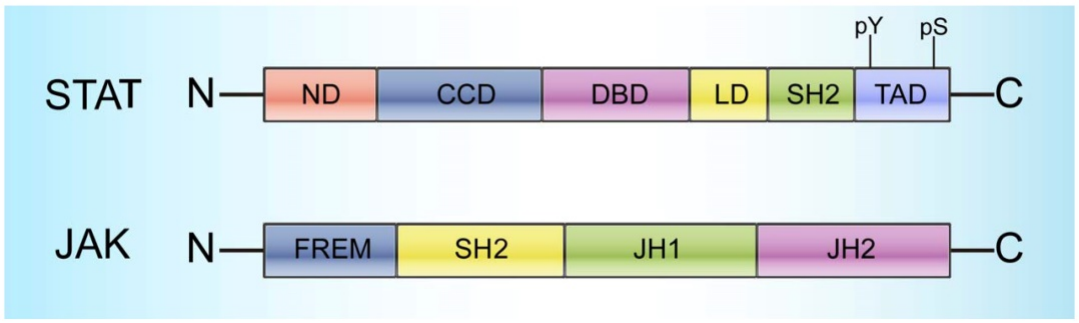
** Structural characteristics of STATs and JAKs.** STATs cover 6 domains: a helical N-terminus domain (ND); a coiled-coil domain (CCD); a central DNA-binding domain (DBD); a linker domain (LD); an Src homology 2 (SH2) domain; and a C-terminal transactivation domain (TAD) with a conserved tyrosine residue at 705 (Y705) and a serine phosphorylation site at 727 (S727). JAKs cover 4 domains: a C-terminal tyrosine kinase domain (JH1); a pseudokinase domain (JH2), which is necessary for maintaining the JAK inactive state and critical for regulating JH1 activity; a FERM (4.1 protein, ezrin, radixin, and moesin) domain; and an SH2 domain.

STATs range in size from 750 to 850 amino acid residues and contain the following 6 conserved domains: 1) a helical N-terminus domain (ND) that can promote binding with DNA and regulate translocation into the nucleus, 2) a coiled-coil domain (CCD) that provides the action site for transcription factors and regulatory proteins, 3) a central DNA-binding domain (DBD) that can determine the specific DNA sequence binding with STATs, 4) a linker domain (LD) that affects DNA binding stability, 5) an Src homology 2 (SH2) domain that recognizes phosphotyrosine residues and is closely related to STAT activation, and 6) a C-terminal transactivation domain (TAD) with a conserved tyrosine residue at position 705 (Tyr-705) and a serine phosphorylation site at 727 (Ser-727) (Figure 1) 22.

Generally, STATs are localized to the cytoplasm and are inactive; stimulation by diverse cytokines as well as growth factors can trigger their subsequent activation 27. Janus activated kinases (JAKs) and other tyrosine kinases can activate STATs by phosphorylating the tyrosine residues on the cytoplasmic domain 28-33. JAKs, a group of receptor-associated cytoplasmic tyrosine kinases, were initially discovered more than 20 years ago and have gained increasing attention due to their critical roles in STAT activation 34-39. Four JAK family members have been identified to date, namely, JAK1, JAK2, JAK3, and TYK2. All four members range in size from 120-140 kDa, and each member has 7 conserved domains consisting of Janus homologies 1-7 (JH1-7) that can be mainly divided into three parts: 1) a C-terminal tyrosine kinase domain (JH1), which can activate JAKs; 2) a pseudokinase domain (JH2), which is necessary to maintain the JAK inactive state and critical for regulating JH1 activity 17, 32, 40; and 3) a FERM (4.1 protein, ezrin, radixin, and moesin) domain and an SH2 domain in the N-terminal region (JH3-7), which are responsible for the interactions between JAKs and various cytokine receptors (Figure 1) 14.

Notably, despite considerable homology, STAT proteins share functional differences. Except for STAT5b, other STAT family members have been demonstrated to participate in atherogenesis in different ways. STAT1, STAT4, and STAT5a have been shown to play an essential role in inflammation during atherosclerosis 8, 41, 42. STAT2 can mediate interferon (IFN) signaling exclusively and then affect atherogenesis 21. STAT6 was found to participate in immune activity and lipid accumulation and thus contributes to atherosclerosis 20. In addition, STAT3 is the most-studied STAT protein in atherosclerosis, not only for its effects on all the above activities but also for its roles in endothelial cell dysfunction.

### 2.2 The structural and functional characteristics of STAT3

As the most conserved protein in the STAT family, STAT3 is composed of the 6 conserved structural domains (ND, CCD, DBD, LD, SH2, and TAD), like other STAT family proteins (Figure 1). Among these domains, both TAD, which has conserved phosphorylation sites at Tyr705 and Ser727, and SH2 can recognize phosphotyrosine residues and are thus closely related to STAT3 activation.

Moreover, STAT3 has been identified as having distinct isoforms, STAT3α, STAT3β, STAT3γ, and STAT3δ, which are considered determinants of its functional heterogeneity 43. Recently, two of the isoforms, STAT3α and STAT3β, showed contrasting effects during the process of atherosclerosis. STAT3α, participating in the mediation of cellular responses to interleukin (IL)-6, is assumed to exert most pro-oncogenic functions. STAT3β, a spliced transcript of full-length STAT3α, replaces the 55 amino acids in TAD with seven different amino acids. In contrast to the effect of STAT3α, STAT3β can not only inhibit inflammatory cytokine synthesis but also promote the expression of certain anti-inflammatory genes 44-46. Mice deficient in both STAT3β and apolipoprotein E (apoE) showed enhanced atherosclerotic plaque formation, most likely due to the unopposed action of STAT3α 47.

STAT3 functions as an essential signal transduction effector protein for cytokine- and hormone-induced pathways that control the development, proliferation or differentiation, and homeostasis of numerous cell types. Like other STATs, STAT3 is mostly activated by phosphorylation of its tyrosine and serine residues via signaling from upstream regulators 48, 49. This phosphorylation event induces dimerization between two STAT3 molecules via reciprocal phosphotyrosine-SH2 interactions. Activated STAT3 dimers then translocate to the nucleus and bind to the consensus promoter sequences of their target genes to initiate transcription. Studies that modulate constitutive STAT3 activation by genetic and pharmacological approaches have verified the critical roles of STAT3 in cell proliferation, apoptosis, angiogenesis, metastasis, and immune responses 50.

However, like phosphorylated STATs, unphosphorylated STAT proteins can also translocate to and prominently exist in the nucleus in various types of mammalian cells in quiescence 51-55. Notably, studies have shown that unphosphorylated STAT3 sustains cytokine-dependent signaling for long periods through a mechanism completely distinct from that used by phosphorylated STAT3 (p-STAT3) 55. Yang and colleagues have shown that the formation of tyrosine-phosphorylated STAT3 (p-STAT3) is stimulated by some gp130-linked cytokines, such as IL-6, accompanied by the activation of many genes, including Stat3 itself 55. The increase in the concentration of unphosphorylated STAT3 drives the expression of a second wave of genes, including RANTES, IL-6, IL-8, MET, and MRAS, which do not respond directly to p-STAT3 55.

Thus, STAT3 functions in two distinct ways in cytokine-dependent transcription: by playing a role in the primary response through formation of p-STAT3 dimers and by playing a secondary role in the complete response through the action of increased amounts of unphosphorylated STAT3.

### 2.3 STAT3 signaling pathway is involved in atherosclerosis

#### 2.3.1 IL-6 cytokine family/JAK2/STAT3 signaling pathway

The IL-6 family of cytokines, including IL-6, leukemia inhibitory factor (LIF), oncostatin M (OSM), neuropoietin (NP), cardiotrophin-1 (CT-1), and ciliary neurotrophic factor (CNTF), are important regulators of JAK2/STAT3 signaling pathway activation in the development of atherosclerosis 34, 56. First, IL-6 family cytokines bind to their corresponding receptor, enabling activation of the expressed signal transductor gp130; this event is followed by the dimerization and phosphorylation of JAK2 and STAT3 57. Next, phosphorylated STAT3 dimers translocate into the nucleus and promote the transcription of target genes, which exert regulatory effects on various activities, such as endothelial cell injury and immune responses 58. H_2_O_2_-induced cell apoptosis and death depend on JAK2 and STAT3 activation 59, 60. Notably, activation of the JAK2/STAT3 pathway is closely associated with the IL-6 cytokine family, which plays an essential role in endothelial cell dysfunction during atherosclerosis. Furthermore, as an important proinflammatory cytokine, IL-6 exerts a profound influence on STAT3-mediated inflammation in atherosclerosis. In vitro studies have demonstrated that after activating the JAK2/STAT3 pathway in vascular endothelial cells, IL-6 upregulates the expression of monocyte chemotactic protein-1 (MCP-1) and exerts a series of proinflammatory effects 61. IL-6 also participates in the differentiation of immune cells through the JAK2/STAT3 signaling pathway. In conclusion, this signaling pathway is involved in the process of atherosclerosis, and its modulation may provide an effective therapeutic option for atherosclerosis.

#### 2.3.2 IL-10/JAK/STAT3 signaling pathway

IL-10 is an immunomodulatory cytokine that has potent anti-inflammatory activity, and its signaling pathway has been well characterized in macrophages and T lymphocytes. IL-10 signaling is mediated via a transmembrane receptor, which consists of a ligand-binding IL-10 receptor1 (IL-10R1) chain and an accessory subunit, IL-10R2 62. The binding of IL-10R1 to IL-10 induces conformational changes and dimerization with IL-10R2, exerting downstream effects 63. Following activation, the IL-10 receptor regulates cellular activity via the JAK-STAT3 signaling pathway 64, resulting in recruitment of the cytoplasmic protein JAK1 followed by phosphorylation of the tyrosine at position 705 in the STAT3 molecule. p-STAT3 forms a homodimer and then translocates to the nucleus to enhance the transcriptional regulation of target genes, such as vascular endothelial growth factor A (VEGF-A), basic fibroblast growth factor-2 and placental growth factor, all major angiogenic factors 65. Loss-of-function approaches targeting the IL-10/STAT3 pathway utilizing genetic and antibody-mediated modalities have shown decreased angiogenesis* in vivo* and* in vitro* 65. Notably, IL-10 expression has been detected in human atherosclerotic plaques, primarily in macrophages 66. Moreover, disruption or cell-specific overexpression of IL-10 in murine models results in modifications of the lesion size, immune cell accumulation, and cell pattern phenotype in the lesion 67-70. Unlike the STAT3 signaling pathway induced by IL-6, the IL-10/JAK/STAT3 signaling pathway has anti-inflammatory functions in macrophages 18. Taken together, these results suggest that this signaling pathway plays a pivotal role in the process of atherosclerosis, especially during macrophage polarization and neovascular proliferation.

#### 2.3.3 Regulation of the STAT3 signaling pathway

STAT3 can be phosphorylated and activated by different factors, among which the activation process through JAKs is the best studied. Various types of stimuli, including cytokines and growth factors, can initiate STAT3 activation. When receptors on the membrane bind with their ligands (e.g., cytokines and growth factors) and dimerize, pairs of JAKs are recruited to the receptor domains and initiate self-activation via either auto- or transphosphorylation 18. Then, the activated JAKs phosphorylate tyrosine residues in the intracellular receptor domains, which can be recognized by the SH2 domain of STAT3 and provide a docking site for STAT3 21. Consequently, the activated JAKs phosphorylate Tyr 705 of STAT3, which contributes to the binding of STAT3 molecules to the receptor domains and the homo- or heterodimerization of STAT3. Next, the homo- or heterodimers of STAT3 translocate to the nucleus, bind to specific sequences on the promoters of target genes and induce the transcription of target genes, such as SOCS3, MCP-1, and VEGF-A 33, 61, 65, 71. The palindromic DNA core motif recognized by STAT3 dimers with 2-fold symmetry is called the GAS element (IFNγ activation site, TTCN_2-4_GAA) 72. The binding affinity to GAS elements varies among STAT proteins. For example, TTC(N)_3_GAA is an optimal variant for STAT1, which prefers binding sites containing an intrasite spacer of three bases (CCG). On the other hand, STAT3, STAT4, and STAT5a/b prefer to bind at sites with 2- to 4-base spacers, and most high-equilibrium binding intensity interactions occur at sites with a 3-base spacer 73, 74 Interestingly, although the phosphorylation of STAT3 is important for its function, the translocation of STAT3 from the cytoplasm to the nucleus may be independent of its phosphorylation status due to the constitutive binding of STAT3 to importin α-3 53. Moreover, STAT3 can also be phosphorylated by some receptor tyrosine kinase (RTKs), including epidermal growth factor receptor (EGFR) and insulin-like growth factor receptor (IGFR), and other nonreceptor kinases, including Src and Abl 7.

Molecules that negatively regulate STAT3, including protein tyrosine phosphatases (PTPs), protein inhibitors of activated STAT (PIASs), and suppressor of cytokine signaling 3 (SOCS3), are critical to prevent its hyperphosphorylation. PTPs regulate the activation of STATs through direct and indirect dephosphorylation of p-STAT3. The indirect regulators, including CD45 and PTP-1B, can downregulate STAT3 activation via dephosphorylation of JAK2 36, 37. SH2-containing protein tyrosine phosphatase (SHP)-1, SHP-2, and PTP receptor T (PTPRT) can dephosphorylate and inactive STAT3 directly 32, 35 Notably, PTPRT specifically dephosphorylates the Tyr705 residue of STAT3 and thus regulates the cellular localization and target gene expression of STAT3 32. Additionally, PIAS3 inhibits the binding of dimerized p-STAT3 to DNA, eventually blocking the target gene transcription of STAT3 17. In particular, cytokines are important for the occurrence and development of atherosclerosis due to their participation in several pivotal signaling pathways associated with atherosclerosis. SOCS3 proteins are considered crucial in the regulation of the cytokine-JAK-STAT3 signaling pathway 75. SOCS3 modulates JAK/STAT3 signaling in a negative feedback loop that utilizes 3 mechanisms: kinase-mediated inhibition of JAKs via the kinase inhibitory region (KIR) domain located at the C-terminus, binding site competition with STATs for initiating JAKs, and gp130 degradation via the SOCS box located at the N-terminus 13, 14. Furthermore, prolonged phosphorylation in SOCS3 gene-deficient mouse macrophages due to IL-6 stimulation suggests that SOCS3 plays an important role in controlling the responses to IL-6 76. Previous studies have demonstrated that the proinflammatory cytokine IL-6 and the anti-inflammatory cytokine IL-10 share the same STAT3 signaling pathway, which induces SOCS3 expression. SOCS3 targets gp130, a receptor for IL6, but not IL-10R, which results in shortened IL-6-driven STAT3 activity, while IL-10-driven STAT3 activation is prolonged 77. The downregulation of JAK-STAT activation secondarily induces the expression of SOCS3, which is a negative regulator of STAT3. This negative feedback through SOCS decreases cellular sensitivity to cytokines and appears to be necessary for suppressing inflammation and cellular proliferation 32, 78.

## 3. Physiopathological roles of STAT3 in atherosclerosis

Atherosclerosis is an intricate process involving multiple cell types, such as endothelial cells and macrophages. In Table 2, we present the essential roles of these cells in the development of atherosclerosis. Atherosclerosis can be triggered by several factors, resulting in dysfunction of the endothelium and accumulation of oxidized low-density lipoproteins (ox-LDLs) in the intima. Ox-LDLs then trigger the expression of adhesion molecules and the secretion of chemokines by endothelial cells, driving monocyte migration and adhesion to the endothelium. Afterwards, the secretion of macrophage colony-stimulating factor (M-CSF) induces the differentiation of monocytes into macrophages, where scavenger receptors recognize and take up highly ox-LDL particles, ultimately leading to foam cell formation 79. Excessive inflammatory and immune responses communicated by these different cell types are driven by inflammatory cytokines and other inflammatory stimuli that promote associated tissue damage and contribute to local inflammation and vascular dysfunction 80-82. Notably, STAT3 can be divided into nuclear STAT3 and mitochondrial STAT3 according to its translocation, and both are believed to play important roles in the development of atherosclerosis, including endothelial cell dysfunction, macrophage polarization, inflammation and immunity.

### 3.1 Endothelial cell dysfunction

Endothelial cells play central roles in the functions of the cardiovascular system 83. Endothelial cell dysfunction causes the accumulation of lipids, inflammatory cells, and coagulation materials as well as vascular smooth muscle cell (VSMC) proliferation, thus promoting atherosclerotic plaque formation 84. Numerous clinical studies have shown that vascular endothelial cell dysfunction is the initiator of and key link to ensuing atherosclerosis 84. Furthermore, endothelial cell dysfunction is closely related to injuries induced by various types of hazards (smoking, hyperlipidemia, oxygen free radicals, etc.) 85. Oxygen free radicals, collectively known as reactive oxygen species (ROS), are the main cause of endothelial cell injury 85. Increased ROS levels are due to an imbalance between their production and elimination and characteristic of oxidative stress. Thus, the injury caused by ROS is classified as oxidative stress injury (OSI) and can increase endothelial permeability, promote leukocyte adhesion, and change endothelial gene expression.

Recently, an increasing number of studies have demonstrated the high activation of the JAK2/STAT3 signaling pathway in the OSI of various cell types (glioma cells, VSMCs, endothelial cells, etc.), which suggests the essential role of this pathway in the modulation of oxidative stress responses 86, 87. Ivan H.W. Ng and colleagues have shown that in a murine embryonic fibroblast (MEF) model system, H_2_O_2_ may cause OSI in endothelial cells through the IL-6/JAK2/STAT3 signaling pathway, the high activation of which allows STAT3 to maintain a phosphorylated state 88. Under normal conditions, cytokine-induced STAT3 phosphorylation is rapid and transient. T cell protein tyrosine phosphatase (TC-PTP) 89 and SOCS3 play important roles in the negative regulation of STAT3 activation, which limits sustained STAT3 phosphorylation. In H_2_O_2_-simulated oxidative stimulation, the activation rate of SOCS3 slows down and the STAT3 phosphatase TC-PTP (TC45) mislocalizes to the cytoplasm, both of which weaken their inhibition of STAT3 phosphorylation 88. These results show that STAT3 does not dephosphorylate in time in instances of endothelial cell injury.

Sustained STAT3 phosphorylation also leads to the abnormal expression of adhesion molecules 90. Adhesion molecules are currently believed to induce inflammation and thus play an important role in the development of atherosclerosis 91. In the early stage of atherosclerosis, adhesion molecules mainly promote monocyte migration, and these cells further adhere to the endothelium 91, 92. As atherosclerosis progresses, adhesion molecules can promote a cascade of mononuclear cells migrating to the lesions, T lymphocyte activation, and interactions between different cells 91. Afterwards, adhesion molecules mediate more cells into plaques, further prompting plaque development and affecting plaque stability 91.

**Figure 2:**
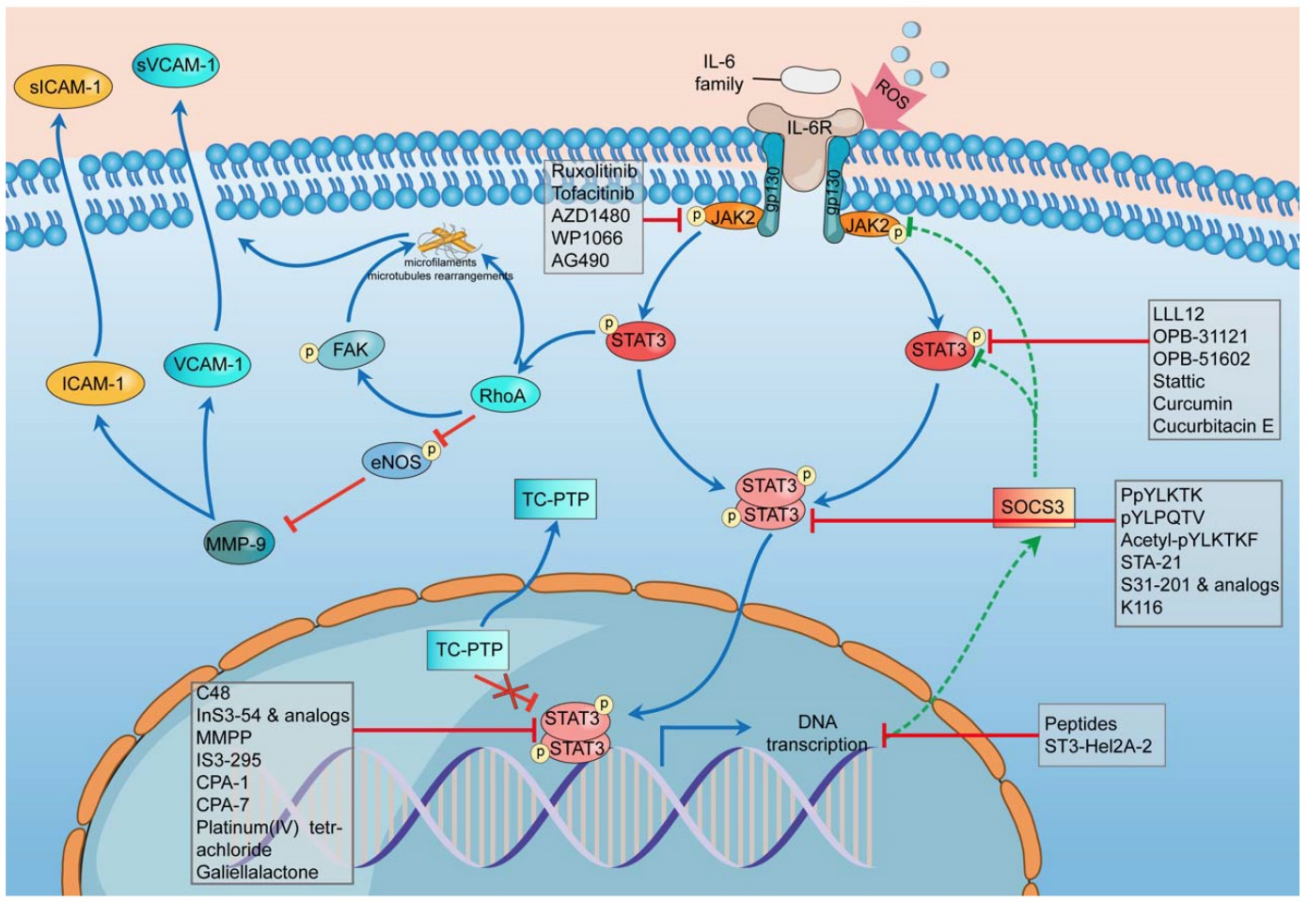
** Schematic diagram of the impact of STAT3 on endothelial cell dysfunction and some STAT3 inhibitors during this process.** In the stimulation of ROS, the transcription rate of SOCS3 slows down, and STAT3 phosphatase TC-PTP (TC45) mislocalises to the cytoplasm, both of which weaken their inhibition of STAT3 phosphorylation and promote the hyperphosphorylation of STAT3. The hyperphosphorylation of STAT3 can activate RhoA which can rearrange the microfilaments and microtubules through its direct effect or through phosphorylating FAK indirectly. RhoA could inhibit the phosphorylation of eNOS thus promoting the effect of MMP-9 to improve the expression of ICAM-1 and VCAM-1. The combination of these mechanisms leads to dysfunction of endothelial cells. The whole process involves five classes of STAT3 inhibitors including indirect inhibitors and direct inhibitors that play an essential role in the phosphorylation, dimerization, DNA-binding, and transcriptional activity of STAT3. ROS, reactive oxygen species; SOCS3, suppressor of cytokine signaling 3; TC-PTP, T-cell protein tyrosine phosphatase; RhoA, Ras homolog gene family member A; eNOS, endothelial nitric oxide synthase; MMP-9, matrix metallopeptidase 9; ICAM-1, intercellular adhesion molecule-1; VCAM-1, vascular cell adhesion molecule-1.

There are 3 main adhesion molecules related to atherosclerosis: intercellular adhesion molecule-1 (ICAM-1), vascular cell adhesion molecule-1 (VCAM-1), and platelet endothelial cell adhesion molecule-1 (PECAM-1) 91. Activation of the IL-6/JAK2/STAT3 signaling pathway can lead to the upregulation and overproduction of ICAM-1 and VCAM-1 in endothelial cells 90. The phosphorylation of STAT3 can activate Ras homolog gene family member A (RhoA), which is an essential regulator, to rearrange the microfilaments and microtubules as well as inhibit the phosphorylation of endothelial nitric oxide synthase (eNOS) 93-95. eNOS phosphorylation can suppress the effect of matrix metallopeptidase 9 (MMP-9), thus downregulating the expression of ICAM-1 and VCAM-1 90. Thus, the suppression of eNOS promotes the expression of ICAM-1 and VCAM-1, which can cause more cells to adhere to and affect the stability of plaques (Figure 2).

In the stimulation of ROS, the transcription rate of SOCS3 slows down, and STAT3 phosphatase TC-PTP (TC45) mislocalizes to the cytoplasm, both of which weaken their inhibition of STAT3 phosphorylation and promote the hyperphosphorylation of STAT3 88. The hyperphosphorylation of STAT3 can activate RhoA, which can rearrange the microfilaments and microtubules through its direct effect or indirectly phosphorylate FAK 96-98. RhoA could inhibit the phosphorylation of eNOS, thus promoting the effect of MMP-9 on improving the expression of ICAM-1 and VCAM-1 96, 99, 100. The combination of these mechanisms leads to endothelial cell dysfunction.

One important manifestation of endothelial cell dysfunction is the abnormal expression of adhesion molecules, which can be caused by ROS through the IL-6/JAK2/STAT3 signaling pathway. This pathway can be highly activated by ROS, leading to the sustained phosphorylation of STAT3 and the further overrelease of ICAM-1 and VCAM-1. Excess adhesion molecules induce inflammatory responses and aggravate endothelial cell dysfunction, thus prompting the development of atherosclerosis 91.

### 3.2 Macrophage polarization

Macrophages are now thought to be present in all stages of atherosclerosis, from the initiation and expansion of lesions to necrosis or rupture, and have become a clinical manifestation of atherosclerosis 101. Macrophages can release a diverse repertoire of inflammatory mediators and construct an inflammatory environment in the atherosclerotic neointima 102, leading to the proximal exacerbation of arterial damage. Notably, the number of macrophages mainly depends on the infiltration and differentiation of monocytes. Thus, monocyte-to-macrophage differentiation is a critical event that accentuates atherosclerosis by promoting an inflammatory environment within the vessel wall 103. STAT3 participates in regulating monocyte-to-macrophage differentiation, and inhibition of STAT3 activity suppresses both inflammation and monocyte-to-macrophage differentiation 103.

Macrophages can respond to various environmental stimuli by adopting one of several functional phenotypes, such as the classically activated macrophage phenotype (M1), the alternatively activated macrophage phenotype (M2), and the Mox macrophage phenotype 104-106. Although they are all secrete inflammatory molecules and factors that further regulate lipoprotein retention, functional differences exist in these different macrophage phenotypes 107. M1 macrophages can produce proinflammatory cytokines, including IL-6, IL-12, and tumor necrosis factors (TNFs), as well as toxic agents, such as nitric oxide (synthesized by inducible NO synthase, iNOS) and free oxidative radicals, thus promoting increased and sustained inflammation and leading to an acute atherothrombotic vascular event 108-113. M2 macrophages are anti-inflammatory macrophages identified by the expression of molecules such as IL-10, arginase 1 (Arg1), and Mrc1 (also known as CD206) and are involved in tissue repair, enhancing plaque stability during atherosclerosis 114-117. Jinjin Cui and colleagues found that the JAK2/STAT3 signaling pathway is involved in M1 polarization 118. Moreover, STAT3 signaling is an essential determinant of the alternative M2 phenotype 65. Targeted inhibition of both IL-10 receptor-mediated signaling and STAT3 activation in macrophages reverses the aging phenotype 65. Given the crucial roles of various macrophage phenotypes in the pathogenesis and progression of atherosclerosis, intervening with macrophage polarization represents a potentially effective therapeutic strategy for atherosclerosis. Therefore, STAT3 may become a potent target to treat atherosclerosis via regulating the polarization of macrophages.

### 3.3 Inflammation

Basic and clinical studies have shown that unchecked chronic inflammation is responsible for many deadly cardiovascular diseases, including atherosclerosis 61. Atherosclerosis is closely correlated with inflammation and exhibits diverse inflammatory behaviors at different stages. In the early stage of atherosclerosis, inflammation is mainly associated with mononuclear macrophage infiltration and increased secretion of proinflammatory cytokines, including IL-6, TNF-α and IL-1β, while in the progressive stage of atherosclerosis, it mainly manifests as massive VSMC proliferation 61, 119, 120. Additionally, studies have found that p-STAT3 mainly localizes in the endothelial nucleus of the inflammatory response region of atherosclerotic plaques but not in the noninflammatory response region, which strongly indicates that STAT3 activation is involved in the atherosclerotic inflammation.

#### 3.3.1 Early stage of atherosclerosis: cytokine secretion

Human atherosclerotic plaques contain various inflammatory cell types, including monocytes, macrophages, and mast cells. Recruitment of these inflammatory cells by adhesion molecules (ICAM-1 and VCAM-1) and E-selectin in endothelial cells is the critical step for inducing the formation of atherosclerotic plaques 121, 122. This stage is characterized by the secretion of some cytokines, mainly IL-6, TNF-α and IL-1β.

TNF-α and IL-1β have been demonstrated to induce the massive IL-6 expression and aggravate vascular inflammation, thus promoting the formation of atherosclerotic plaques 123. Together with TNF-α and IL-1β, IL-6 is thought to participate in B cell maturation and T cell differentiation and drive acute inflammatory response 61. However, sustained IL-6 production plays a role in the chronic low-level inflammation associated with atherosclerosis 124, 125. Moreover, IL-6 has been detected in atherosclerotic plaques, and it mainly functions by the JAK/STAT3 signaling pathway in atherosclerosis 61, 119. Binding of the IL-6/IL-6R complex to gp130 results in activation of the JAK/STAT3 signaling pathway and induction of proinflammatory IL-6-responsive genes, including MCP-1 and ICAM-1, inducing a series of proinflammatory effects 61, 71.

#### 3.3.2 Progressive stage of atherosclerosis: VSMC proliferation and migration

Excessive proliferation and migration of abnormal VSMCs are major causes of atherosclerosis development 126. In response to atherogenic factors secreted by endothelial cells and leukocytes, including platelet-derived growth factor (PDGF)-BB, endothelin-1, thrombin, and IL-1, VSMCs proliferate massively, migrate into the intima, and modify the inflammatory phenotype in atherosclerosis and thus are the main cell type associated with arterial intimal thickening 127. VSMCs can be classified into two major phenotypes: one includes fully differentiated, contractile cells responsible for vasodilation and vasoconstriction, and the other consists of migratory, proliferative cells activated during growth or injury 128. Notably, switching of the two phenotypes from the former to the latter is known to be vital for stable plaque formation 128. However, VSMC apoptosis leads to drastic vessel remodeling, with increased inflammation and coagulation and thinning of the fibrous cap, making the plaque more prone to rupture 129, 130. Thus, reducing the excessive proliferation of VSMCs, improving their functions, and preventing their apoptosis could substantially slow the development of atherosclerosis.

Accumulating studies have demonstrated that activated STAT3 participates in the proliferation of VSMCs. After inhibiting STAT3 in human aortic VSMCs cells, cell proliferation is obviously reduced, while activating STAT3 via LIF enhances cell proliferation 131. Angiotensin (Ang)-II promotes oxidative stress and mediates VSMC proliferation, thus playing an essential role during this period. The JAK2/STAT3 signaling pathway, a well-known contributor of oxidative stress, is closely associated with the Ang-II type 1 receptor and nuclear transcriptional changes, such as the activated transcription of early growth response genes, eventually resulting in VSMC proliferation 132-135. Moreover, a study has verified that Ang-converting enzyme 2 (ACE2), a specific Ang-II-degrading enzyme, can attenuate VSMC proliferation via suppressing the activation of the JAK2/STAT3/SOCS3 signaling pathway 136.

The transcript levels of PDGF-BB in human atherosclerotic plaques are increased compared to those in normal arteries 137. Moreover, as the most potent mitogen, PDGF-BB has been shown to induce VSMC migration and intimal hyperplasia in the arteries of carotid injury model rats, mice, and porcine* in vivo* 138. Importantly, accumulating evidence supports an important role for the activation of STAT3 in PDGF-BB-induced VSMC proliferation and migration 139. A previous study demonstrated that PDGF-BB-induced VSMC motility requires activation of the JAK2/STAT3 signaling pathway 140. PDGF-BB can promote the tyrosine phosphorylation of JAK2 and STAT3 in a time-dependent manner 140. However, the dominant negative mutant-dependent suppression of JAK2 and STAT3 can block PDGF-BB-induced VSMC migration 140. These results indicate that the JAK2/STAT3 pathway plays an important role in PDGF-BB-induced VSMC migration.

Phenotypic switching is also a pivotal step underlying many VSMC-related pathological conditions, especially atherosclerosis. Liao and colleagues have demonstrated that the JAK/STAT3 signaling pathway is a central regulator of the VSMC phenotypic switch 131. These researchers found that knockdown of endogenous STAT3 enhances the VSMC contractile phenotype by promoting the association of the myocardin/serum response factor-CArG complex. In contrast, the activated STAT3 signaling pathway suppresses the expression of VSMC-specific contractile protein genes and is thus positively correlated with the synthetic VSMC phenotype 131. Therefore, the phenotypic switch of VSMCs can be controlled by modulation of the JAK/STAT3 signaling pathway. Inhibition of STAT3 activation can prevent the VSMC contractile phenotype from switching to the inflammatory phenotype, eventually slowing the progression of atherosclerosis.

### 3.4 Immunity

Immune cells, especially CD4^+^ T cells, play a central role in the physiological process of atherosclerosis based on their vital roles in the cellular immune network. CD4^+^ T cells are divided into various cell types according to different effectors, including Th1, Th2, Th9, Th17, Th22, T follicular helper (Tfh), and regulatory T (Treg) cells 141.

After the discovery of Th17 cells, several groups addressed their potential contribution to atherogenesis 142. Moreover, elevated numbers of Th17 cells, which produce the proinflammatory molecule IL-17A, are associated with autoimmune diseases and have been observed in atherosclerotic lesions 143. Recently, Th17 cell processes were verified to be closely related to the occurrence and development of atherosclerosis, and STAT3 is the key regulator of Th17 cell differentiation through IL-6 induction 121, 144. The combined stimulation of transforming growth factor (TGF)-β and IL-6 can initiate the differentiation of CD4+ T cells into Th17 cells in mice 145. IL-6 upregulates the expression of IL-21 through the STAT3 pathway, which then increases the expression of the IL-23 receptor and the retinoic acid-related orphan receptor (ROR)γt 146, 147. In cooperation with STAT3, RORγt promotes the expression of IL-17 and inhibits the expression of forkhead transcription factor p3 (Foxp3) 148, 149. In the early stage of atherosclerosis, IL-6 inhibits Foxp3 and promotes the expression of RORγt by activating STAT3 150. In the intermediate stages, IL-21 secreted by the cell itself promotes the expression of the RORγt and IL-23 receptors through activation of STAT3, resulting in a positive feedback effect 151-153. During the later stages, IL-23 also promotes the expression of IL-22 and inhibits the effects of IL-10 through STAT3, enabling the complete differentiation of Th17 cells 150. Furthermore, IL-6-mediated mitochondrial Ca^2+^ sustains the production of two cytokines (IL-21 and IL-4) known to be regulated by IL-6 in CD4^+^ cells 154. Thus, mitochondrial STAT3 can sustain prolonged cytokine production and contribute to the differentiation of CD4^+^ T cells in atherosclerosis.

Treg cells provide protection against autoimmunity and are regarded as promising targets of clinical therapies to treat various diseases caused by autoimmunity, including atherosclerosis 155, 156. Treg cells can modulate several processes involved in the development of atherosclerosis. For example, Tregs can inhibit proatherogenic T cells, dendritic cell (DC) activation and migration, macrophage inflammation, foam cell formation, EC activation and affect cholesterol metabolism 157. STAT3 mutations disenable Treg cells to produce IL-17, indicating that IL-17 secretion and Treg cell functions depend on STAT3 158. Additionally, the Th17/Treg cell imbalance plays key roles in atherosclerosis in apoE^(-/-)^ mice 159. STAT3 can regulate the Th17/Treg ratio, which is closely related to the amount of IL-6 and the number of Treg cells 160.

A comparison of DCs in different intima showed that the atherosclerotic intima contains significantly more DCs than the normal intima, suggesting that DCs play an active role in the initial stage of atherosclerosis 161. Studies have demonstrated that DCs distributed in atherosclerotic lesions are mainly responsible for antigen presentation and T cell activation in the lesion 102. IL-6 secreted by mature DCs plays an important role in the differentiation of CD4^+^ T cells into Th17 cells by the IL-6/JAK2/STAT3 pathway 162. Additionally, T cell activation and Th cell polarization are shaped by costimulatory molecule engagement and exposure to a specific cytokine milieu, with Th1 cells critically depending on IL-12 secretion from DCs. Studies have shown that STAT3 inhibits IL-12 cytokine production in DCs and increases STAT3 mRNA expression but decreases IL-12 transcript expression in advance compared with that in early lesions 163-165. These data indicate that STAT3 in DCs plays an essential role in the development of atherosclerotic lesions.

### 3.5 STAT3 in mitochondria

In addition to its well-established roles in the nucleus during the progression of atherosclerosis, STAT3 is also present in mitochondria and contributes to the regulation of the electron transport chain (ETC) activity. As a major source of cellular ROS, mitochondrial-derived reactive oxygen species (mtROS) are natural byproducts of the ETC 166. Importantly, several lines of evidence indicate that excessive mtROS-induced oxidative damage occurs in atherosclerotic lesions in both animal models and humans, indicating that excessive mtROS is associated with atherosclerosis progression 166. Although not required for mitochondrial function, mitochondrial STAT3 can directly interact with ETC complexes, enhancing ETC activities independent of nuclear activity and eventually contributing to enhanced oxidative phosphorylation (OXPHOS) and ATP production 18, 167. Additionally, mitochondrial STAT3 is a regulator of ETC and Ca^2+^ homeostasis and affects the mitochondrial production of ATP and ROS, thus playing a crucial role in atherosclerosis.

## 4. Physiopathological roles of other STATs in atherosclerosis

In addition to STAT3, other STATs, especially STAT1 and STAT2, also play essential roles in atherosclerosis. STAT1 has been identified as a regulator of foam cell formation and atherosclerotic lesion development in an intraperitoneal inflamemation model and an atherosclerosis-susceptible bone marrow transplantation mouse model 168. Moreover, increased STAT1 activity results in VSMC proliferation and neointimal hyperplasia, while deficient STAT1 in the bone transplantation mouse model reduces macrophage apoptosis and plaque necrosis 169, 170. STAT1 also elevates the expression of chemokines and promotes oxidative stress and tissue injury by stimulating the NADPH oxidase gene and protein expression 171, 172. Moreover, apoF is a sialoglycoprotein component of high-density lipoproteins (HDL) and LDL fractions in human serum. Although the exact role of STAT2 in atherosclerosis has not been reported, genetic manipulation of the apoF/Stat2 locus supports an important role for STAT2-dependent type I IFN signaling and gene expression in atherosclerosis 173.

## 5. Inhibitors

The above findings provide convincing evidence that STAT3 can be a novel therapeutic target for atherosclerosis. Therefore, intensive efforts have been devoted to developing STAT3 inhibitors. Currently, there are two strategies for inhibiting the STAT3 signaling pathway: indirect and direct STAT3 inhibitors. The former strategy can block molecules upstream of the STAT3 signaling pathway and indirectly inhibit the signal transduction functions of STAT3, for example, by inhibiting the function of JAKs, Src, Abl, and Lyn 174-181. The latter strategy can be divided into several types according to different target domains, including the SH2 domain, DBD, ND, and TAD. Here, we summarize important STAT3 inhibitors targeting the JAK2/STAT3 signaling pathway and STAT3 structural domains.

### 5.1 Indirect inhibitors

Since the substitution of valine with phenylalanine at amino acid 617 (V617F) within the JH2 'kinase-like' domain of JAK2 was demonstrated to result in an overactivation of JAK2, inhibitors targeting JAK2 specifically have become the focus of studies. Over the years, numerous JAK2 inhibitors have been designed, including ruxolitinib, tofacitinib, AG490, AZD1480, SB1578, and WP1066 182-185. These inhibitors inhibit the JAK2/STAT3 signaling pathway in a similar way, which indicates that they may function by suppressing immune and inflammatory responses during the development of atherosclerosis. Notably, ruxolitinib and tofacitinib have been approved by the FDA for the treatment of myelofibrosis and rheumatoid arthritis 186. However, their potential application in the treatment of atherosclerosis has not been investigated. AG490 has been widely used as a JAK2 inhibitor in cardiovascular research but has no clinical application 187. The most promising novel substance is WP1066, which achieved outstanding results in the treatment of malignant and vascular diseases. In preclinical studies, WP1066 was not only shown to significantly impact the prevention of tumor angiogenesis but also to successfully prevent neointima formation and to contribute to plaque stability in atherosclerosis 182, 183, 188. However, these kinase inhibitors, which are similar in structure to those of other kinases, play a major role in the enzymatic catalytic center and thus often exert off-target effects. Recently, flavonoids have become a research hotspot due to their potential to inhibit STAT3 activity. Surprisingly, researchers have demonstrated that tricin, a bioflavonoid expressed at significantly high levels in rice bran of Njavara, can significantly inhibit the activation of both STAT1 and STAT3 via the downregulation of upstream phosphorylation enzymes such as JAK1 and JAK2, exerting a potent anti-inflammatory effect 189. Additionally, the heterocyclic small molecules naringenin and flavone exhibit the novel combined functions of inhibiting IL-6-promoted STAT3 and inducing SOCS3 in vascular endothelial cells. Nevertheless, their mechanism of inhibition contrasts with that of structurally related traditional JAK inhibitors, which inhibit both IL-6-promoted STAT3 activation and SOCS3 induction independently 61. Taken together, these results suggest that indirect inhibitory strategies through traditional JAK inhibitors have relatively large adverse reactions and need further improvement. However, emerging natural inhibitors, such as tricin, are indirect STAT3 inhibitors that could be used to treat atherosclerosis and have fewer side effects than previously used inhibitors. Moreover, the newly identified mechanism by which flavonoids (naringenin and flavone) inhibit STAT3 may provide an avenue for developing novel therapies for atherosclerosis based on the induction of negative STAT3 regulators.

### 5.2 Direct inhibitors

#### 5.2.1 Inhibitors targeting the SH2 domain

There has been recent interest in the direct inhibition of STAT3, and this approach has mainly focused on targeting the SH2 domain 190, 191. The SH2 domain, which contains a pocket that binds to another STAT3 protein, is an important domain by which STAT3 maintains its biological functions. After phosphorylation at the Tyr705 site of the STAT3 protein, the SH2 domain binds two activated STAT3 monomers to form a dimer, translocates into the nucleus, and binds to DNA to initiate the transcription of specific target genes. Inhibition of the SH2 domain results in impairment of not only STAT3 activation and dimerization but also the subsequent nuclear translocation and expression of STAT3-regulated genes.

Peptide and peptide-like STAT3 inhibitors, including PpYLKTK (P: proline, L: leucine, K: lysine, T: threonine) 192, pYLPQTV (V: valine) 193, and acetyl-pYLKTKF (F: phenylalanine) 194, mainly target the SH2 domain. These inhibitors can impede the binding of two STAT3 monomers, thus preventing the dimerization and nuclear translocation of STAT3 proteins and inhibiting the binding of STAT3 to DNA.

STA-21 (deoxytetrangomycin) and its structurally optimized analog LLL12 can bind to the SH2 domain, blocking STAT3 dimerization and DNA binding 160, 195. Furthermore, OPB-31121 and OPB-51602 have been demonstrated to interact with the SH2 domain of STAT3 with high affinity 196. Cell culture assays showed that OPB-31121 and OPB-51602 inhibited the phosphorylation of both Tyr705 and Ser727. Moreover, both of these proteins were able to uniquely bind to the STAT3 SH2 domain, which does not overlap with the binding site for other STAT3 inhibitors. Their distant binding pocket and potent affinity for STAT3 SH2 make OPB-31121 and OPB-51602 the most promising candidates for further development 197. Phase I/II studies on the use of STA-21 for the treatment of psoriatic lesions and on OPB-31121 and OPB-51602 for the treatment of advanced cancers have been completed, revealing that all of these compounds are effective inhibitors of STAT3 phosphorylation and have acceptable safety profiles. Although they have not been clinically used for atherosclerosis treatment, further clinical development of these compounds is expected in the future. Other notable small-molecule STAT3 inhibitors, including Stattic, S3I-201 and S3I-201 analogs, bind to the STAT3 SH2 domain and inhibit STAT3 activity. Notably, molecular docking studies show that S3I-201, LLL12, and Stattic have highly conserved pTyr-SH2 binding pockets, allowing the primary targeting of STATs but not STAT3 specifically 72.

Another group of inhibitors targeting the SH2 domain of STAT3 are derivatives from natural compounds. Curcumin, a naturally derived phytochemical from plants such as turmeric (*Curcuma longa*), has many pharmacological activities, including antioxidant, anticarcinogenic, antiobesity, antiarthritic, analgesic, hepatoprotective, antiangiogenesis, and anti-inflammatory properties 198-213. Previous studies have shown that curcumin may exert antiatherosclerotic effects by regulating a broad spectrum of factors linked to inflammation and oxidative stress 214-216. Additionally, curcumin affects lipid metabolism, subsequently alleviating hyperlipidemia and atherosclerosis 201. Notably, curcumin has been shown to inhibit STAT3 phosphorylation and block the STAT3 signaling pathway in various types of cell types 217, 218. Based on this new insight, curcumin also represents a potential therapeutic option for atherosclerosis as an effective STAT3 inhibitor. Resveratrol is another essential natural inhibitor of STAT3 that has the ability to attenuate some detrimental atherosclerotic processes, such as transportation of LDL, oxidation of lipids, and invasion of macrophages 219, 220. Furthermore, resveratrol has been tested in preclinical studies and clinical trials for the treatment of atherosclerosis 219. Preclinical studies showed promising results for the use of resveratrol to treat atherosclerosis, revealing that its antioxidant and anti-inflammatory properties may mediate its antiatherogenic effect 219. Nevertheless, clinical trials investigating the effect of resveratrol on the plasma lipid profiles in human subjects have not been as clear 219. Notably, in some experimental conditions, curcumin and resveratrol may activate rather than inhibit STAT3 functions, and the acute activation of STAT3 by these drugs during stroke and myocardial infarction promotes cell survival. Additionally, cucurbitacin E 221, alantolactone 222, piperlongumine 223, and silibinin 224 all bind the STAT3 SH2 domain and thus play essential roles in STAT3 inhibition. However, most of these agents are not STAT3-specific, as they are capable of binding the SH2 domains of all STAT proteins. Smaller compounds, such as resveratrol, were shown to predominantly target only the pTyr-binding cavity, while higher molecular weight compounds, including curcumin and cucurbitacin E, bound additional SH2 cavities 72.

These inhibitors play important roles in endothelial cell dysfunction, anti-inflammation and immunity, which means that they may be promising treatment agents for atherosclerosis. Unfortunately, these inhibitors still have some negative qualities. Although peptides and peptide-like compounds have high biological activities, they are easily metabolically inactivated and have low bioavailability, which limits their further development in medical arenas. Additionally, most inhibitors targeting the SH2 domain are not STAT3-specific, which makes it difficult to rule out the roles of other STATs in atherosclerosis 72. Furthermore, due to the difficulty of developing small molecules capable of disrupting protein-protein interactions over a large surface area that still retain drug-like properties, only a limited number of SH2 domain inhibitors have reached preclinical and clinical trials 225-228.

#### 5.2.2 Inhibitors targeting the DBD

The DBD is an important binding region between the STAT3 protein and DNA and has a relatively high specificity. This domain can induce the expression of target genes via the binding of STAT3 to target gene promoters.

To date, few small molecules have been reported as STAT3 DBD inhibitors due to the lack of an efficient in vitro screening assay, hindering the drug discovery process. C48 was first found to alkylate glutathione sulfhydryl on a STAT3 cysteine (C468), a unique ammonia acid residue in the STAT3 DBD region 229. Afterwards, two small-molecule inhibitors targeting the DBD, inS3-54 and inS3-54a18, were shown to inhibit the STAT3 dimer from binding DNA 230. Of the analogs tested, inS3-54a18, A26, and A69 were identified to be more active than the parent inS3-54. Moreover, several platinum compounds, including IS3-295, CPA-1, CPA-7, and platinum (IV) tetrachloride, have the same effect 231, 232. Galiellalactone, a natural product, was identified as an inhibitor of the DNA-binding activity of STAT3, thus impeding STAT3 downstream gene expression through the IL-6/STAT3 signaling pathway 233. A study found that galiellalactone likely binds the DBD of STAT3 to block the activity of STAT3 both in vitro and in vivo 234. Collectively, inhibitors targeting the DBD mainly affect the endothelial cell dysfunction in atherosclerosis. By suppressing the expression of target genes, these inhibitors can help return SOCS3 expression to normal levels. Thus, the sustained phosphorylation of STAT3 can be controlled by SOCS3, and endothelial cell dysfunction can be reduced.

Because DBDs are flat with similarities among different isoforms of the same transcription factor family, they are generally considered “undruggable”. Although substantial progress has been made regarding inhibitors targeting the DBD of STAT3, these inhibitors still face challenges similar to those related to the therapeutic utilization of the SH2 domain described above.

#### 5.2.3 Inhibitors targeting other domains

Inhibitors targeting ND and TAD can mediate the binding of STAT3 dimers and regulate DNA transcription, which may be useful for treating atherosclerosis by suppressing endothelial cell dysfunction, inflammation, and abnormal immune cell differentiation. Researchers have synthesized a highly selective STAT3 ND inhibitor, ST3-Hel2A-2, that effectively activates the expression of the proapoptotic gene CHOP and thus induces apoptosis in tumor cells 235. Moreover, researchers found that in addition to the SH2 domain of the STAT3 protein, some sites (D171, N175, Q202 and M213) in the TAD domain can inhibit STAT3 dimerization and regulate DNA transcription. From the TAD, researchers were able to precisely screen for the allosteric active small molecule K116 that can bind to TAD and inhibit the activity of STAT3 236.

## 6. Implication of STAT3 in atherosclerosis

As we have illustrated above, individuals with abnormal STAT3 activity are prone to atherosclerosis. Thus, targeting the STAT3 pathway has become a popular therapeutic approach in the treatment of atherosclerosis. Currently, an increasing number of STAT3 inhibitors have been identified and shown effects on not only inflammatory or proliferative diseases but also on diseases caused by vascular cell function 237-241.

Inhibitors approved by the FDA, including ruxolitinib and tofacitinib, suggest that STAT3-inhibiting strategies may offer promising developments in clinical fields 237-241. Recently, Johnson et al. provided the first evidence that inhibitors of STAT3 activation protect against Ang-II-induced oxidative stress, endothelial dysfunction, and hypertension in mice 242. Oxidative stress and endothelial dysfunction are key steps in the process of atherosclerosis, as we have presented above, which means that STAT3 inhibitors may block the development of atherosclerosis and thus be novel therapeutic options. WP1066, which showed outstanding results for treating vascular diseases, is expected to be the most promising novel therapeutic agent in the near future. In preclinical studies, WP1066 successfully prevented neointima formation and contributed to plaque stability in atherosclerosis 182, 183, 188. The same results have been found for a number of STAT3-inhibiting natural compounds, including capsaicin, curcumin, cryptotanshinone and resveratrol, which have numerous clinical implications 227. Collectively, STAT3 inhibitors, which block oxidative stress and vascular dysfunction, could serve as therapeutic agents for atherosclerosis.

## 7. Conclusions and perspectives

Previously, substantial evidence has provided support for the hypothesis that STAT3 is a prominent regulator of various cancers, ischemic injury, and obesity. However, the role of STAT3 in the regulation of atherosclerosis has not been clearly illustrated, and atherosclerosis is still a threat to humans. In this review, we provided a basic overview of STAT3 and showed its pathological roles in atherosclerosis. After summarizing previous studies, we illustrated how aberrant STAT3 activation contributes to endothelial cell dysfunction, macrophage polarization, inflammation, and immunity and may thus become an essential modulator during atherosclerosis. Therefore, inhibitors targeting the STAT3 signaling pathway are expected to be promising therapeutic methods for the treatment of atherosclerosis. We then summarized the current STAT3 inhibitors, which are indirect and direct inhibitors, among which we may find novel therapies for atherosclerosis.

However, the road leading from STAT3 inhibitors to mature therapies for atherosclerosis remains long, and the therapeutic application of STAT3 inhibitors has some limitations. First, STAT3 inhibitors are toxic and potent. From a security standpoint, natural products, including curcumin and resveratrol, are good candidates for STAT3 inhibition because of their noticeable dose-limiting toxicity 243. However, their relatively poor bioavailability and potency limit their application in the treatment of various diseases, including cancers and atherosclerosis 243. As described above, several STAT3 inhibitors, especially those targeting the SH2 domains, are not STAT3-specific, which weakens their effectiveness to some extent. Moreover, somatic mutations in STAT3, which may affect its binding site, render the inhibitors described above ineffective and affect the outcomes of clinical studies. Unfortunately, studies considering somatic mutations are complex, and no study has yet addressed this problem. In the future, understanding the relationship between somatic mutations and STAT3 inhibitors and identifying more effective inhibitors presents a difficult challenge. Although numerous direct STAT3 inhibitors have been developed and several STAT3 inhibitors have completed Phase I/II clinical trials, targeting STAT3 as an atherosclerosis therapy remains frustratingly elusive. Therefore, there is an urgent need to reassess ongoing strategies and to develop clinically useful drugs. Additionally, the occurrence and development of atherosclerosis is a complicated process in which STAT3 plays a critical but not solo role, and the inhibitors that simply target the STAT3 pathway may thus not be sufficiently potent. Therefore, combinations of STAT3 inhibitors with other targeted therapeutics may be promising. To determine the most promising combinations, we must have a thorough understanding of the pathways participating in atherosclerosis and the interactions among them.

Moreover, current STAT3 inhibitors are not sensitive to the different isoforms of STAT3. Four isoforms of STAT3 have been identified, STAT3α, STAT3β, STAT3γ, and STAT3δ, which all have different functions. STAT3α and STAT3β have been verified to have contrasting effects during the process of atherosclerosis 237, which suggests that STAT3 inhibitors must discriminate among distinct isoforms to preserve their functions.

Finally, it is important to consider any negative bodily effects when holistically inhibiting STAT3 activation, as STAT3 is found to have positive effects on some processes, including cardiac protection, wound healing, and angiogenesis 244-247. Early studies have shown that activation of STAT3 plays important roles in myocardial recovery from myocarditis-induced damage in adult mammalian hearts 244. Furthermore, activated STAT proteins, including STAT3, upregulate cardioprotective genes, such as *bcl-xL* and *VEGF*, playing an important role in the maintenance of cardiac function 245, 247-249. STAT family proteins also reportedly function as regulators of angiogenic growth factors, and their signaling in cardiac myocytes can control vessel growth during cardiac remodeling 247. Moreover, increased expression of STAT3 in combination with other factors, including decreased miR-17-5p expression, can lead to wound healing 246. However, when inhibiting cardiac STAT3 by its dominant negative form in zebrafish, cardiomyocyte proliferation after ventricular amputation is decreased by ~80%, resulting in insufficient heart regeneration 244, 250. Importantly, cardiomyocyte-specific ablation of the STAT3 gene was shown to suppress the frequency of cycling cardiomyocytes in the recovery period without influencing the inflammatory status, resulting in impaired tissue repair and cardiac dysfunction 244. Therapies aimed at systemic STAT3 inhibition under conditions that are already associated with reduced STAT3 activity in organs may further promote heart failure 38. Thus, for the treatment of single atherosclerotic lesions, a local application might be a favorable option given that local application using drug-eluting stents or balloons may exert potent effects with negligible systemic side effects.

The time to adopt treatment is also essential, as earlier treatment times are correlated with increased effectiveness. However, due to low penetrance in diseased regions, identifying the stage of atherosclerosis is difficult. Thus, the effectiveness of such therapies also depends on advances in diagnostic methods. After overcoming these challenges, the inhibition of STAT3 has the potential to be a valuable therapeutic strategy for atherosclerosis.

## Figures and Tables

**Table 1:** Inhibitors of STAT3

**Indirect inhibitors of STAT3**					
**Classification**	**Inhibitors**	**Year**	**Target site**	**Mode of targeting STAT3**	**Reference**
**Small-molecule inhibitors**	Ruxolitinib	2012	JAK1/2	Phosphorylation	182, 186
	Tofacitinib	2013	JAK3	Phosphorylation	185
	AZD1480	2011	JAK1/2	Phosphorylation	184
	SB1578	2010	JAK2	Unknown	182
	WP1066	2010	JAK2	Phosphorylation	183
	AG490	1996	JAK2	Phosphorylation	187
	Naringenin	2013	SOCS3	Unknown	61
	Flavone	2013	SOCS3	Unknown	61
**Natural inhibitors**	Tricin	2014	JAK1/2	Phosphorylation	189
**Direct inhibitors targeting the SH2 domain of STAT3**					
**Classification**	**Inhibitors**	**Year**	**Target site**	**Mode of targeting STAT3**	**Reference**
**Peptides and peptide-like inhibitors**	PpYLKTK	2001	SH2	Dimerization	192
	pYLPQTV	2003	SH2	Dimerization	193
	Acetyl-pYLKTKF	2007	SH2	Dimerization	194
**Small-molecule inhibitors**	STA-21	2009	SH2	Dimerization	195
	LLL12	2012	SH2	Phosphorylation	160
	OPB-31121	2013	SH2	Phosphorylation	196
	OPB-51602	2015	SH2	Phosphorylation	197
	Stattic	2006	SH2	Phosphorylation	196
	S31-201 & analogs	2007	SH2	Dimerization	196
**Natural inhibitors**	Curcumin	2014	SH2	Phosphorylation	198-200
	Cucurbitacin E	2010	SH2	Phosphorylation	221
	Alantolactone	2015	SH2	Unknown	222
	Cryptotanshinone	2009	SH2	Unknown	227
	Piperlongumine	2015	SH2	Unknown	223
	Silibinin	2015	SH2	Unknown	224
**Direct inhibitors targeting DBD and other domains of STAT3**					
**Classification**	**Inhibitors**	**Year**	**Target site**	**Mode of targeting STAT3**	**Reference**
**Small-molecule inhibitors**	C48	2011	DBD	DNA binding	229
	InS3-54 & analogs	2014	DBD	DNA binding	222, 230
	MMPP	2017	DBD	DNA binding	222
**Platinum compounds**	IS3-295	2005	DBD	DNA binding	231, 232
	CPA-1	2004	DBD	DNA binding	231, 232
	CPA-7	2004	DBD	DNA binding	231, 232
	Platinum (IV) tetrachloride	2004	DBD	DNA binding	231, 232
**Natural inhibitors**	Galiellalactone	2014	DBD	DNA binding	233, 234
	Peptides	2007	ND	Transcriptional activity	192
	ST3-Hel2A-2	2013	ND	Transcriptional activity	235
	K116	2018	TAD	Dimerization	236

**Table 2:** Roles of different cell types in the development of atherosclerosis

Cell type	Stage of atherosclerosis in which they participate	Trigger factor	Effects of STAT3	Effect on the development of atherosclerosis	Reference
Endothelial cells	Mainly in the initial stage of atherosclerosis	ROS or sustained STAT3 phosphorylation	Causing endothelial cell dysfunction, including activation of RhoA, rearrangement of microfilaments and microtubules, upregulation and overexpression of adhesion molecules	Vascular endothelial cell dysfunction is the initiator of and key link to ensuing atherosclerosis. Promoting accumulation of lipids, adhesion of inflammatory cells, and proliferation of VSMCs.	84, 88, 90, 91, 93-95
Macrophages	Almost in all stages of atherosclerosis	TNF-α; IL-1β	Affecting the number of macrophages in the microenvironment by regulating monocyte-to-macrophage differentiation, participating in M1 phenotype polarization	Expressing scavenger receptors, taking up high ox-LDL particles, leading to foam cell formation. Macrophages can adopt different functional phenotypes according to the atherosclerotic environment. M1 is a proinflammatory phenotype that leads to atherosclerosis, while M2 is an anti-inflammatory phenotype that maintains the stability of atherosclerotic lesions	65, 79, 101, 103, 108-113
VSMCs	In the progressive stage of atherosclerosis	Ang-II; atherogenic factors including PDGF-BB, endothelin-1, thrombin, and IL-1	Promoting Ang-II- and PDGF-induced VSMC proliferation and migration; regulating the phenotypic switch of VSMCs	Excessive proliferation and migration of abnormal VSMCs are major causes of the development of cardiovascular diseases, including atherosclerosis; excessive synthesis of extracellular matrix, thickening of arterial walls, and development of atherosclerotic plaques	126-128, 131-136, 140
CD4^+^ T cells	In all stages of atherosclerosis	IL-6	Sustaining prolonged cytokine production; contributing to the differentiation of CD4^+^ T cells; regulating the Th17/Treg ratio	CD4^+^ T cells, especially Th1, Th17, and Treg cells, participate throughout the entire atherosclerosis process, playing important roles in the rupture of atherosclerotic plaques and Th17/Treg cell imbalance, leading to atherosclerosis	121, 142-144, 154, 157-160
DCs	In the late stage of atherosclerosis	IL-6; IL-12	Inhibiting IL-12 cytokine production	Promoting antigen presentation and T cell activation in the lesion; Th cell polarization by IL-12 secretion	161-165
